# The power, potential, benefits, and challenges of implementing high-throughput sequencing in food safety systems

**DOI:** 10.1038/s41538-022-00150-6

**Published:** 2022-08-16

**Authors:** Behzad Imanian, John Donaghy, Tim Jackson, Sanjay Gummalla, Balasubramanian Ganesan, Robert C. Baker, Matthew Henderson, Emily K. Butler, Yingying Hong, Brendan Ring, Clare Thorp, Ramin Khaksar, Mansour Samadpour, Kahlil A. Lawless, Iain MacLaren-Lee, Heather A. Carleton, Renmao Tian, Wei Zhang, Jason Wan

**Affiliations:** 1 Illinois Institute of Technology, Chicago, IL USA; 2 Société des Produits Nestlé S.A, Vevey, Vaud Switzerland; 3 Driscoll’s, Watsonville, CA USA; 4 American Frozen Food Institute, Bethesda, MD USA; 5 Mars, Incorporated, McLean, VA USA; 6 Land O’Frost, Lansing, IL USA; 7 Abbott Nutrition, Columbus, OH USA; 8 Creme Global, Dublin, Dublin, Ireland; 9 Clear Labs, San Carlos, CA USA; 10 Institute for Environmental Health, Inc., Seattle, Washington, USA; 11 Illumina, Inc, San Diego, CA USA; 12 Oxford Nanopore Technologies, Oxford, UK; 13 Centers for Disease Control and Prevention, Atlanta, GA USA; 14 Present Address: Jackson Group Consulting, San Jose, CA USA; 15 Present Address: Oaklare Strategic Scientific Consulting LLC, Fairfax Station, VA USA

**Keywords:** Comparative genomics, Bacterial genetics

## Abstract

The development and application of modern sequencing technologies have led to many new improvements in food safety and public health. With unprecedented resolution and big data, high-throughput sequencing (HTS) has enabled food safety specialists to sequence marker genes, whole genomes, and transcriptomes of microorganisms almost in real-time. These data reveal not only the identity of a pathogen or an organism of interest in the food supply but its virulence potential and functional characteristics. HTS of amplicons, allow better characterization of the microbial communities associated with food and the environment. New and powerful bioinformatics tools, algorithms, and machine learning allow for development of new models to predict and tackle important events such as foodborne disease outbreaks. Despite its potential, the integration of HTS into current food safety systems is far from complete. Government agencies have embraced this new technology, and use it for disease diagnostics, food safety inspections, and outbreak investigations. However, adoption and application of HTS by the food industry have been comparatively slow, sporadic, and fragmented. Incorporation of HTS by food manufacturers in their food safety programs could reinforce the design and verification of effectiveness of control measures by providing greater insight into the characteristics, origin, relatedness, and evolution of microorganisms in our foods and environment. Here, we discuss this new technology, its power, and potential. A brief history of implementation by public health agencies is presented, as are the benefits and challenges for the food industry, and its future in the context of food safety.

## Introduction

The global food supply chain is an intricate system of numerous interconnected producers, processors, distributors, consumers, and regulators, each playing a critical role in the farm to fork continuum. Population growth, globalization, and consumers’ changing expectations have added extra layers of complexity to this system and more challenges to its management. Continuous attention, proactive surveillance, and vigilance are increasingly required to detect, identify, and mitigate the risks of foodborne pathogens, spoilage organisms, allergens, and other food safety hazards that may enter food at any stage from production to consumption. These risks are evident in the history of outbreaks associated with domestic and imported foods. Incorporating scientific and technological advances in food safety systems is a crucial strategy to keep pace with escalating complexity of food systems and expanding risks that threaten their functionality and safety.

The past two decades have witnessed the convergence of diverse technical fields, resulting in novel molecular instruments with enhanced accuracy, precision, speed, and throughput, ushering us into an era of “big data”. The impacts of these advances are being felt widely, profoundly, and often globally. While we have shown proficiency in discovery and innovation, our adoption and integration of these technological upgrades have been slow and less systematic across various scientific disciplines. The human genome project is an excellent example of an insightful, international, multidisciplinary, transparent, and successful undertaking^1,2^. Among the many stated and achieved goals of the project was the development of technology that revolutionized genomics via high-throughput or next-generation sequencing (HTS/NGS). In this review, we have used the terms HTS and NGS interchangeably to refer to all the post-first-generation (or post-Sanger) sequencing technology and platforms that are distinguished from their predecessors, especially by their significantly higher throughputs or the number of sequences and data they produce. We have conducted a thorough search of the scientific literature, using different search engines to find, review, and reference the most relevant and recent publications on the top journals and academic research databases using words and phrases such as HTS, NGS, sequencing technology, microbial pathogens, foodborne bacteria and many more pertaining to the use of HTS/NGS by the governmental agencies and food industry. In addition, we have carefully searched the world wide web (e.g., the official websites of governmental agencies, biotechnology companies, various laboratories and consulting and analytical enterprises that use HTS) for relevant information. Promoting the use and implementation of this technology and its many applications in our food safety system will improve our food safety inspections, strengthen our responses to food-borne disease outbreaks and, more revolutionarily, enhance our capabilities for preventive measures^3^. Here, with these potential impacts in mind, we present our brief review of the recent advances in HTS and its impacts on food industry and three U.S. federal agencies: Centers for Disease Control and Prevention (CDC), Food and Drug Administration (FDA), and the Department of Agriculture’s Food Safety and Inspection Service (USDA FSIS).

## HTS development by biotechnology companies

Advances in HTS technology by biotechnology companies have been tied to their ability to secure funding, and to monitor, identify, acquire, and incorporate new relevant scientific and technological innovations from many fields, notably in biochemistry, optics, nanofluidics, nanofabrication, engineering, and computer science. This has often been achieved through acquisitions of other smaller companies established by academics. Illumina, PacBio, and Oxford Nanopore are a few success stories that reveal a common trend towards smaller, smarter, cheaper, and scalable instruments with enhanced chemistry and shortened library preparation time, higher quality and longer reads and higher throughput that can be processed by sophisticated bioinformatic and visualization tools, thus simplifying the user experience^4–6^.

Illumina sequencers use Sequencing by synthesis (SBS). The molecular clustering technology enhanced accuracy of base calling due to stronger signals and reduced cost of system optics^6^. The introduction of complementary metal oxide semiconductor^7^ in conjunction with a patterned flow cell with billions of evenly spaced nanowells led to further improvement in the light detection emitted by incorporating labeled nucleotides to the growing synthesized DNA chain^8^. Switching from 4-channel to 2-channel or 2-image/cycle SBS shortened the data processing and sequencing time^9^. Use of the Nextera transposon-mediated library prep reduced the time, cost, and complexity of sample and library preparation^10^. One of the latest Illumina sequencers, the NovaSeq 6000 System, can produce 16–20 billion paired-end reads of 150 bp, 85% of which have a Q30 or higher quality (≤1 error in 1000 bp) amounting to a maximum throughput of 3TB (https://www.illumina.com/systems/sequencing-platforms/novaseq/specifications.html). New improved algorithms have led to better consensus accuracy and higher read quality. To date, over 20,000 sequencing systems from Illumina are installed and supported globally https://www.illumina.com/company/news-center/press-releases/press-release-details.html?newsid=e3ad6d4e-fcbe-4a0d-ac1d-5a3996d1bfd8.

PacBio also built upon a combination of advances in semiconductor processing, photonics and biotechnology^11^. These innovations have significantly increased the length of the analyzed sequences^12^. With improved quality, the latest PacBio instruments provide a reliable solution for sequencing highly repetitive and difficult-to-sequence regions of a genome. PacBio improved the quality of sequences by refining its signal detection technique through zero-mode waveguides (ZMWs). The flow cell contains many ZMWs that facilitate and enhance detection of the light emitted from labeled nucleotides added to the growing DNA strands in all nanowells simultaneously. This allows a real-time sequencing unlike Illumina instruments, where sequencing occurs in cycles interrupted by the base calling process. However, the real-time sequencing in both PacBio and Oxford Nanopore sequencing does not read individual nucleotides unlike in Illumina sequencing. Thus, there is still a possibility for homopolymer errors^13^, but they are being constantly reduced through algorithmic improvements for base-calling. The density of ZMWs in newer PacBio sequencers is significantly increased reaching eight million in one of the recent instruments, thus providing higher throughput. Latest PacBio instrument can produce up to 4,000,000 reads of a median average of 15 kb with a consensus accuracy of up to 99.999% (https://www.pacb.com/technology/hifi-sequencing/sequel-system/; https://www.pacb.com/literature/smrt-sequencing-brochure-delivering-highly-accurate-long-reads-to-drive-discovery-in-life-science/).

Oxford Nanopore is best known for building the first hand-held sequencer and longer reads^14–16^. Its technology is mainly based on advances in strand sequencing in a modified nanopore and an enzyme motor to control DNA movement through the pore^17,18^. Instead of the emitted light, the ionic current within the pore is detected and measured. The protein nanopore is set in an electrically resistant polymer membrane, and the ionic current is passed through the nanopore by setting a voltage across this membrane. As a strand of DNA is captured and fed through the pore, a change in electrical current occurs which can be detected by extremely sensitive sensors and interpreted as a DNA sequence. Interestingly, the same methodology allows direct sequencing of RNA, DNA with modified bases (e.g., methylated), proteins and small molecules^19,20^. Additionally, the read length is determined by the length of the DNA molecule passing through the pore, so it is effectively unlimited. Oxford Nanopore has greatly improved the output of sequence data by increasing the density of pores and sensor channels on their flow cells. Oxford Nanopore’s PrometION 48, at full capacity, can theoretically produce 14 TB of sequence data with a read median length of 30 kb, and the best read quality achieved so far is 99.3% (https://nanoporetech.com/q20plus-chemistry; https://nanoporetech.com/products/specifications).

With increased throughput, the three companies needed to address significant data processing and analytical challenges. Following acquisitions of software and data management companies, Illumina developed its BaseSpace cloud-computing platform. There, users can stream and analyze their data with numerous click-and-compute applications (https://www.illumina.com/products/by-type/informatics-products/basespace-sequence-hub/apps.html) and share the results without the need for investment in computing infrastructure.

To address the high error rates in the first generation of long reads, PacBio employed computational approaches and algorithms. PacBio’s latest instruments can now perform heavy-duty computations and sequence longer HiFi reads obviating the need for thousands of CPU hours on a high-performance computer (HPC) (https://www.pacb.com/wp-content/uploads/Pacific-Biosciences-New-Sequel-IIe-System-Puts-Focus-on-High-Read-Accuracy.pdf). This is especially helpful for small and medium-sized laboratories and companies that would have to invest on separate HPCs for their analyses. Most recently, PacBio has updated their microbial whole genome assembly pipeline to address the unique challenges of assembling bacterial genomes with circular chromosomes and plasmids of varying sizes.

Oxford Nanopore has also updated its instrument control software, computational capabilities and analytical tools internally or in collaboration with NVIDIA https://nanoporetech.com/about-us/news/oxford-nanopore-and-nvidia-collaborate-partner-dgx-ai-compute-system-ultra-high. The recent gains in sequence accuracy have come through iterative chemistry improvements, reengineering of the protein pore structure and especially via continuous enhancements in the machine learning methods used to interpret the raw signal to bases (ACGT). Interestingly, all three companies have formed their own user communities that actively share experiences, provide support for each other and contribute to the development of new analytical tools.

Other laboratories, analytical and consulting firms have increasingly contributed to the development and improvement of HTS technology, marketing untapped new applications and services for various sectors of the economy including the food industry. Some are developing better and cheaper reagents for sample and library preparation (e.g., Qiagen, Agilent, Zymo, Thermo Fisher Scientific, IEH). A few have streamlined the process flow and improved reproducibility (e.g., Roche, Qiagen, Thermo Fisher Scientific, Clear Labs). Many are actively investigating new or existing commercial HTS applications to replace traditional methods reliant on binary (yes/no) and/or culture-based tests (e.g., IEH, Clear Labs, CosmosID^21^). Additionally, many proprietary or public sequence databases have been created, customized and curated for specific purposes addressing authenticity, food spoilage and adulteration, genetic modifications (e.g., GMO), microbial composition, detection of a particular microorganism, speciation and serotyping (e.g., Creme Global, Clear Labs, CosmosID, IEH^22^). Others focus on developing analytical tools (e.g., Qiagen’s CLC, Biomerieux’s Bionumerics, Genevia Technologies), while some are exploring how machine learning (ML) and artificial intelligence (AI) can be utilized to data mine sequences and associated metadata to provide predictability and preventive capability (e.g., Creme Global, Clear Labs, IEH).

## Adoption and use of HTS by U.S. governmental agencies

One important factor for the success and rising use of HTS technology has been its adoption and practice by various governmental agencies for applications in health care and other areas including food safety.

In the United States, CDC, FDA and USDA FSIS work together with state agencies to protect public health and food safety. To this end, agencies work to identify and implement cost-effective advances in technology, such as HTS and its Whole Genome Sequencing (WGS) application, to rapidly detect and investigate foodborne outbreaks of domestic and international origin^23^. The decreasing cost of WGS (https://www.genome.gov/about-genomics/fact-sheets/Sequencing-Human-Genome-cost) coupled with its superior discriminatory power compared to previous generations of “gold standard” methods such as Pulsed-Field Gel Electrophoresis (PFGE) has resulted in a transition to WGS across regulatory agencies in the United States and several other countries. Unlike PFGE, WGS can provide subtyping information as well as serotype, virulence gene profile, and antibiotic resistance determinants. Additionally, WGS provides more precision in determining potential outbreak clusters than PFGE^24^.

In 1996, CDC founded PulseNet, a national molecular surveillance network for foodborne disease. PulseNet currently consists of 83 states, local, and federal public health laboratories. Also, laboratories in 86 countries contribute to PulseNet International. PulseNet uses standardized molecular subtyping techniques and analytical tools to characterize strains of foodborne bacteria and detect potential outbreak clusters by identifying genetically related pathogen fingerprints examined across the country. In collaboration with FDA and USDA FSIS, CDC compares molecular fingerprints generated from pathogens isolated from patients to those of food, animal, and environment to identify potential sources of foodborne disease outbreaks. The establishment of PulseNet^25^ and the use of PFGE to identify foodborne pathogens revolutionized outbreaks’ detection and investigations by these agencies and networks. PFGE outperformed nearly all previous approaches and remained the main method for traceback for two decades prior to WGS.

As an initial proof of concept for using WGS, in 2010, CDC used WGS to characterize the *Vibrio cholerae* strains from an outbreak in Haiti^26,27^. In 2013, FDA, USDA, National Center for Biotechnology Information (NCBI) and an initial pilot group of ten states collaborated on a project to use WGS for surveillance of *Listeria monocytogenes*^28^. Afterwards, PulseNet began to use WGS routinely to characterize isolates associated with outbreaks especially *Salmonella*, E. coli, *Campylobacter*, *Vibrio*, and *Shigella*. In 2019, PulseNet fully transitioned to WGS as the new gold standard for molecular subtyping^29^. CDC funding to the District of Columbia, Puerto Rico and all 50 states facilitated this transition https://www.cdc.gov/pulsenet/pathogens/wgs.html. Since 2013, USDA FSIS also developed its own WGS capabilities, and now sequences all pathogenic isolates routinely, submitting the data to NCBI in real-time^30^.

In 2013, the FDA’s Center for Food Safety and Applied Nutrition launched an integrated network of federal and state laboratories, called the GenomeTrakr (GT). In collaboration with NCBI, the network established its public database, also called GT, for WGS data from foodborne and environmental bacterial pathogens^30,31^. The FDA has also been working closely with the Office of Regulatory Affairs to implement full integration of FDA’s GT network into the Laboratory Flexible Funding Model (LFFM). With LFFM and other incentives, the GT network has expanded to 54 federal, state health and university laboratories in the U.S. and 21 laboratories in 10 other countries. The GT database has been steadily growing, and now holds WGS data for over 752,000 isolates, with over 13,000 new entries each month (https://www.fda.gov/food/whole-genome-sequencing-wgs-program/genometrakr-fast-facts). The FDA has also developed GalaxyTrakr, a distributed analysis tool to process public health WGS data for non-bioinformaticians^32^. The use and implementation of HTS/WGS by governmental Agencies have improved our response time and quality to any outbreak^33–36^.

Through consortia such as the Genomics for Food Safety and other venues, the three federal agencies work closely to harmonize their efforts to improve and standardize WGS laboratory and analytical protocols. PulseNet and GT laboratory networks, for example, have developed a harmonized proficiency test exercise, conducted annually, on the same set of strains, following the same standard operating procedure for genomic data collection^37^, and soon, for data analysis. While different agencies, for historical and practical reasons, use slightly different analytical tools and pipelines, the end results have been scrutinized, compared and found to be concordant^28,30,38,39^.

There are initiatives to promote the transparency and timely public sharing of the WGS data generated from foodborne pathogens in clinical, environmental, and food samples^40,41^. The product of one such initiative for open data sharing is NCBI’s Pathogen Detection (PD) database (https://www.ncbi.nlm.nih.gov/pathogens/) of WGS data for numerous foodborne pathogens. All isolates in PD are grouped into genetically similar clusters within 50 single nucleotide polymorphism to facilitate outbreak detection and traceback. As the PD database of foodborne pathogens grows, so does its value in monitoring pathogens’ characteristics, distribution, relatedness, and evolution. These, in turn, improve understanding of a pathogen’s reservoirs and routes of contamination, along with the root causes of foodborne diseases and potential preventive measures.

In addition to WGS, the federal agencies are actively experimenting with other HTS applications. For example, amplicon sequencing (e.g., 16s rDNA, ITS, ITS1, ITS2) can provide information about the composition of the microbial community in a mixed sample at a given place and time^42^. The CDC is developing an approach to use HTS to amplify thousands of targets in a metagenomics sample that are then sequenced, and the data are used for subtyping. Also, the shotgun sequencing of total DNA or RNA of the same sample can provide further information by unraveling the genomics of nearly all the individual members of the community and their metabolic interactions^43^. These additional applications generate information that can be utilized by public health, food safety, and other investigators^44^.

Many clinical and diagnostic laboratories are moving toward culture-independent diagnostic tests (CIDTs) for the identification of foodborne bacteria from patient samples. With CIDT an isolate is not required for identification of the pathogen, and clinical laboratories can forward a residual stool specimen to a public health laboratory, which meet the reporting requirements^45^. PulseNet is now evaluating metagenomics approaches for generating molecular fingerprints directly from specimens, including patient samples. Metagenomics approaches present challenges like low-signal-to-noise ratio when a small amount of target DNA, for example, of a pathogen like shiga toxin-producing *E. coli* (STEC), is present in a sample along with a large amount of background DNA from commensal, nonpathogenic *E. coli*, human and non-human—e.g., in a stool sample. For PulseNet’s evaluation of metagenomics approaches, PulseNet is using amplicon sequencing to amplify thousands of subtyping targets associated with foodborne pathogens in the stool and enrichment techniques to enrich the pathogen DNA in the metagenomic sample. Metagenomics also presents opportunities to discover new pathogens or bacteria not commonly associated with foodborne outbreaks, infections due to multiple strains, or potential outbreak sources by evaluating food DNA in stool specimens^46^.

Working with public and private food industry stakeholders and their academic partners, the FDA is also developing enhanced protocols and procedures for the environmental sampling of STEC and other enteric foodborne pathogens. The combination of HTS technology, on-the-farm environmental pathogen surveillance, and open sharing of those genomes will help stakeholders discover linkages among isolates that may lead to a better understanding of agricultural sources and routes of contamination, and preventive controls to reduce contamination of ready-to-eat produce.

The high-throughput shotgun sequencing of environmental samples is now the best approach to generate comprehensive genomic data about food safety or spoilage-related microorganisms (bacterial, eukaryotic, viral) in a microbial community^47–49^. Such data generated and accumulated over time provide valuable information about each microbial community and its members with a time-place stamp. These data, in conjunction with relevant metadata representing abiotic factors (e.g., temperature, pressure, acidity, salinity turbidity) and information about associated significant events (e.g., disease outbreaks, spoilage, and recalls) will be the resources for future artificial intelligence (AI) systems, helping us predict, prevent or resolve such problems.

## Current HTS use, challenges, and opportunities in the food industry

Food producers, processors, distributors, and consumers each have high-stakes roles in ensuring food safety and preventing foodborne disease outbreaks. According to USDA, foodborne illnesses cost over $15.6 billion annually, and CDC estimates that 1 in 6 Americans gets sick and about 3000 die every year from consuming contaminated food (https://www.cdc.gov/foodsafety/cdc-and-food-safety.html#:~:text=Foodborne%20illness%20is%20a%20common,than%20%2415.6%20billion%20each%20year).

Two main factors contribute to the food industry’s interest in implementing HTS. First, HTS has the potential to improve control measure design and verification and/or reduce food safety management costs. Second, WGS is adopted by governmental agencies, and the use of WGS and other HTS applications are gaining prominence in public and private sectors. The publication of general requirements and guidance for WGS by the International Organization for Standardization^50^ and the statement by the European Food Safety Authority on the WGS requirement for microorganisms intentionally used in the food chain^51^ are testimonials to HTS/WGS growing importance in governmental circles.

The food industry recognizes that HTS, especially WGS, provides the highest discriminatory power to identify and distinguish pathogens, and could improve the tracing of contamination sources, develop an understanding of their origin and characteristics and thus better predict and prevent persistence or future contamination events. Incorporating the precision provided by WGS into root cause analyses could enable more rapid identification and better targeting of corrective actions, enhancing productivity and lowering costs. The industry has begun to appreciate more the value of metagenomic applications of HTS for environmental monitoring and its predictive value (https://www.cremeglobal.com/wp-content/uploads/2019/12/Case-Study-SAFE.pdf) and to understand factory/ingredient microbial ecology and investigate quality and spoilage incidents where the causative agents are unknown or reasons for recurrence are not understood. However, many food companies have not actively embraced the latest technology for applicable operational use. Even where it has been applied, many enlist the use of third-party providers for technical support when and if required.

A number of global food manufacturers have invested in the technology and created internal infrastructure to apply HTS in-house, and actively or intermittently use HTS^52^ for various purposes including source tracking and/or environmental surveys and monitoring. Their numbers have grown slowly but steadily especially in the past few years. Nestlé and Mars are among the first to invest in the infrastructure for and experiment with HTS. Research and development (R&D) is an active area where these companies are testing and evaluating the multifaceted use of this technology. Such evaluations are conducted, for example, to compare the accuracy of different sequencing platforms for serotype predictions, to investigate laboratory cross-contaminations^53^, to validate sequence workflows, to understand pathogenic genetic responses to heat stress and source tracking^54,55^, and to catalog, characterize and quantify foodborne pathogenic bacteria and the related bacterial communities along the food supply chain^56^.

While much has been articulated by the Agencies and academics regarding the benefits, use, and implementation of HTS, few publications have focused on the benefits and practical applications of HTS from a food industry perspective. In one review, the industry authors discussed the importance of WGS in international tracking of foodborne diseases, its benefits over all previous subtyping methods/tools, and its extensive use and implementation by the Agencies. They encouraged food companies to learn more about WGS, its benefits, and obstacles^52^. However, compared to the older subtyping methods, they also concluded that the added cost of WGS limited its application to routine testing, and the potential benefits did not justify the cost for routine programs. The authors recommended its application for occasions where microbiological verification programs identify persistent or recurring issues/problems and are unable to determine root causes. A closer evaluation of the practices, challenges, and concerns of the food industry about this new technology may help to identify and demonstrate benefits that would justify reinforcing existing food safety management tools with various HTS applications.

Sequencing instruments, new IT plus computational capabilities, and bioinformatic expertise are among the essentials for the use of HTS that many food manufacturers do not have. A lack of internal resources to conduct traditional microbiological or modern molecular testing (e.g., HTS/WGS) is often remedied by using external third-party laboratories. However, this could limit access to the historical isolates, which can be helpful to determine root causes. Thus, the benefits from historical source tracking or trend analysis could not be realized. Larger food manufacturers with in-house analytical facilities could make the most of retained isolates for in-depth investigations, whereas small and medium-sized manufacturers may only perform isolated studies for a particular incident. If they conduct a “costly” investigation with a limited number of isolates, and the results do not assist with a root cause discovery, they may not perceive the value and be able to justify the cost for future use. Also, food manufacturers point out that the process can be slow and is performed “after the event” leading them to use it sporadically, not proactively.

Apart from source tracking, food companies that have adopted WGS have reported other practical applications: 1) Discovering the occurrence and extent of laboratory cross-contamination during analytics; 2) Uncovering hygienic engineering limitations of some equipment, which act as harborage sites for pathogens; 3) Identifying raw materials from the global supply chains that can lead to cross-contamination in manufacturing factory environments. The use of WGS to identify instances of laboratory cross-contamination could reduce waste from investigative costs or affected products if false-positive results occur from cross-contamination^53^. When such events are identified, they also bring about better awareness of good laboratory practices, segregated testing of sensitive from less sensitive materials, and improvements in the use of uniquely identifiable positive controls.

A food safety management system requires appropriate and robust analytical verification tools and methods that involve testing raw materials, the production environment, and finished products. The food industry currently utilizes a host of traditional culture, immunological, and molecular-based diagnostics that are readily available, relatively inexpensive, and rapid. These tests provide sufficient information to meet both regulatory and supply chain requirements and verify the production of food under hygienic conditions. The uptake and use of WGS by a food manufacturer, therefore, will depend on several factors inter alia: the number of occasions the plant has experienced deviations in their verification; the perceived cost-benefit of a WGS investigation; WGS accessibility in-house or through a third party; the type of the pathogen and whether cheaper identification tools are available; and more critically, the potential negative regulatory and legal implications. The higher precision of WGS demands clear rules of engagement and communications to alleviate the apprehensions of using it actively. These rules should address: (1) Retrospective outbreak investigation and linking of new isolates with past illnesses; (2) Use and purpose of metadata associated with the sequences; (3) The incongruity or agreement between the WGS and related epidemiological findings.

There is a wide agreement that the increased precision HTS methods provide supplements or alternatives to the traditional but predominantly microbiological and molecular tools and methods. Further innovations by biotechnology companies, new entrepreneurial approaches to cost reduction and market expansion, along with expanding use of the HTS applications by regulatory agencies and academia will help the industry discover practical and cost-effective applications of the technology in food safety management systems. A new and comprehensive cost-benefit analysis is needed that includes the values of new infrastructure, skilled employees, loss prevention, and predictive capabilities that may not currently exist.

## Data Availability

All the data used for this manuscript are publicly available and are referenced in the text and/or in the References section.
